# PCIR: a database of Plant Chloroplast Inverted Repeats

**DOI:** 10.1093/database/baz127

**Published:** 2019-11-07

**Authors:** Rui Zhang, Fangfang Ge, Huayang Li, Yudong Chen, Ying Zhao, Ying Gao, Zhiguo Liu, Long Yang

**Affiliations:** Agricultural Big-Data Research Center and College of Plant Protection, Shandong Agricultural University, Tai’an 271018, China

## Abstract

Inverted repeats (IRs) serve as potential biomarkers for genomic instability, DNA replication and other genetic processes. However, little information can be found in databases to help researchers recognize potential IR nucleotides, explore junction sites and annotate related functional genes. Plant Chloroplast Inverted Repeats (PCIR) is an interactive, web-based platform containing various sequenced chloroplast genomes that enables detection, searching and visualization of large-scale detailed information on IRs. PCIR contains many datasets, including 21 433 IRs, 113 plants chloroplast genomes, 16 948 functional genes and 21 659 visual maps. This database offers an online prediction tool for detecting IRs based on DNA sequences. PCIR can also analyze phylogenetic relationships using IR information among different species and provide users with high-quality marker maps. This database will be a valuable resource for IR distribution patterns, related genes and architectural features.

## Introduction

Inverted repeats (IRs) are DNA secondary structures that consist of six or more nucleotides in reverse complementary sequence orientation (1,2). Shortly after the β-helix structure of DNA was proposed by Watson and Crick (3), the existence of IRs was recognized to account for some biological processes of cells (4,5). IRs are distributed abundantly in a variety of eukaryote and prokaryote plastids (2). When the transcription or replication processes occurring, two strands separating and IRs might produce with nucleotides transiting from inter-strand to intra-strand base pairing. When the two DNA strands are separated during transcription or replication processes, IRs can result in nucleotides transitioning from inter-strand to intra-strand base pairing. If the symmetric region has a complementary interaction in only one strand, a hairpin structure is formed. However, the extrusion of two opposite hairpins in both strands can lead to the formation of a cruciform structure (4,6). This biological mechanism has been proposed to account for the role of long IRs (>500 bp) in stimulating recurrent chromosomal translocations and generating chromosomally unbalanced offspring (7–9). In contrast, short IRs (≤30 bp), which are enriched in the human genome (7), can prevent DNA replication forks to stimulate DNA double-strand breakage (10).

IRs play significant roles in regulating genetic instability (11), gene splicing (12, 13) and replication stalling (14). *In vivo*, the most distinct property of IRs is their tendency to decrease genomic stability in various kinds of organisms. The repeat length, motif composition, sequence position and genetic background of IRs all have crucial roles in influencing genetic stability (4). In eukaryotes, endogenous transcripts, especially transcribed IRs, produce small RNAs to trigger gene splicing (15–18). In bacteria, replication forks stop at IR positions depending on the length of the central spacer (19).

Many tools are available to detect IRs, such as Mfold (20), findIR (21), detectIR (22), IRF (23), Emboss (24) and Palindrome analyser (25). However, most of these tools require high-level computing skills and lack visualization functions. Mfold is an available online tool (http://unafold.rna.albany.edu), but it can only detect IRs with the input sequences no more than 9000 bases. FindIR and DetectIR are two comprehensive tools for detecting IRs, but they are used based on the commercial software MATLAB. Another tool allowing IR detection is IRF, which only contains a command line version of the IR algorithm. Besides these, Emboss as a standalone application for UNIX can detect IR structures without any presentation and visualization abilities. Palindrome analyser is also a web tool that can be easily performed to detect IRs, but they limit the amount of IRs that can be shown to 5000, and only the registered users can upload/import sequences and store results. In addition, as increasing numbers of chloroplast genomes have been sequenced, IRs have been explored in the chloroplast genomes of many species, such as *Quercus acutissima* (26), *Aster tataricus* (27), *Carnegiea gigantea* (28), *Strobilanthes cusia* (29), *Colobanthus apetalus* (30) and *Sanguisorba* (31). Therefore, a comprehensive platform for IR analysis in plant chloroplast genomes is urgently needed.

In this study, the Plant Chloroplast Inverted Repeats (PCIR) database was established to provide a reliable platform to identify, annotate and analyze IRs in the cpDNAs of sequenced plants and to provide a series of webpages to display IR junction features. PCIR should greatly contribute to research on the distribution, characteristics and functions of IRs.

## Materials and Methods

### Chloroplast genome sequencing data

The chloroplast genome datasets of 113 plant species were screened from the GenBank repository of NCBI and downloaded without annotations (Table S1). The minimum criteria for these genomes in the PCIR relational database were as follows:

(i) all plant genomes from public published studies;
(ii) the genome sequence is complete, not partial; and
(iii) the chloroplast genome reads length is at least 50 kb.

These standards ensured that reads from plant samples were sequenced with similar experimental techniques, and search queries could be established using consistent criteria to minimize variations in reads between samples.

### Identification of IRs

An IR usually comprises two DNA sequences of equal length and includes bases in a reverse complementary direction that form a palindrome. Based on IR structures, a pipeline composed of Perl scripts was used to scan each chloroplast genome. The parameters of IR motif length were set to 6–30 and no mismatches or gaps were allowed among the sequences. The IR positions were also identified by this pipeline.

### Phylogenetic analysis and annotation

The phylogenetic relationships of the 113 species were determined from their complete chloroplast genome sequences. HomBlocks (32) was used to align nucleic acid sequences, and MEGA X (33) was used to generate a phylogenetic neighbor-joining (NJ) tree with 1000 bootstrap replicates. Furthermore, each cpDNA genome was annotated using the online web tool Dual Organellar GenoMe Annotator (34) with default parameters. The circular organelle genome maps of these species were drawn with OGDRAW (35), and the linear IR junction sites were predicted using IRScope (36).

### Database implementation

**Figure 1:**
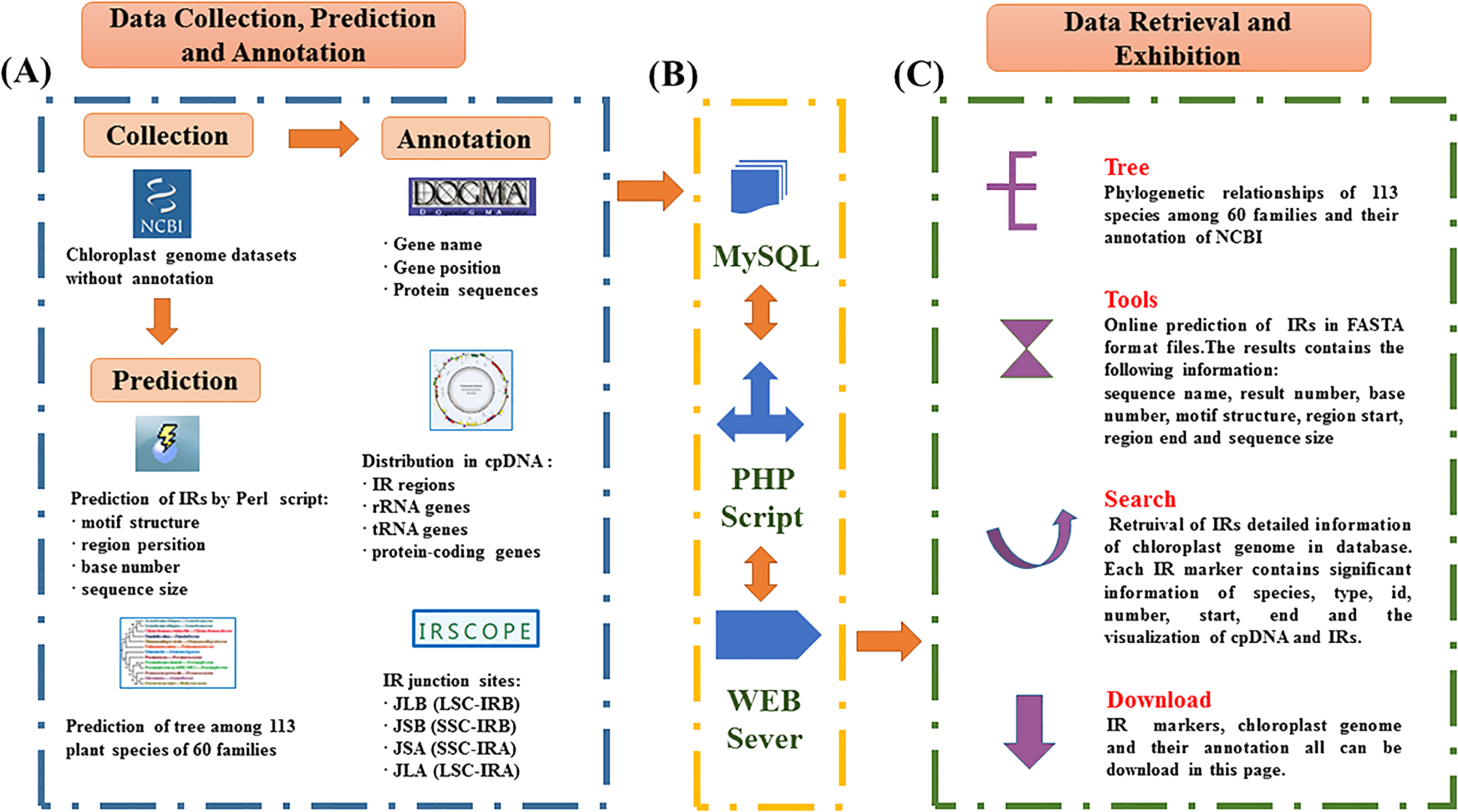
Schematic overview of IR database. (**A**) Data sources of PCIR. (**B**) Workflow of PCIR. (**C**) Data acquisition in the platform.

The database was constructed based on a LAMP framework (Centos 6.5, Apache 2.4.27, MySQL 14.4, Xampp 3.2.2 and ThinkPHP 3.2.3/PHP7.1.9). All IR data were imported into MySQL for efficient management, searching and display. The web-friendly interface was based on HTML5 and PHP. Perl and Java scripts were used to enhance the Common Gateway Interface programs. A complete workflow portraying the content exhibition procedures used in setting up PCIR is shown in Figure 1.

**Table 1:** Numbers, frequencies and proportion of IRs according to size

**IR size**	**Amount in dataset**	**IR/KB**	**Proportion of all**	**IR size**	**Amount in dataset**	**IR/KB**	**Proportion of all**
6	7260	0.4214	0.3387	19	283	0.0164	0.0132
7	3468	0.2013	0.1618	20	250	0.0145	0.0117
8	2069	0.1201	0.0965	21	231	0.0134	0.0108
9	1398	0.0811	0.0652	22	216	0.0125	0.0101
10	1107	0.0643	0.0516	23	191	0.0111	0.0089
11	917	0.0532	0.0428	24	174	0.0101	0.0081
12	685	0.0398	0.0320	25	148	0.0086	0.0069
13	567	0.0329	0.0265	26	130	0.0075	0.0060
14	497	0.0288	0.0232	27	108	0.0063	0.0050
15	437	0.0254	0.0204	28	95	0.0055	0.0044
16	382	0.0222	0.0178	29	84	0.0049	0.0039
17	349	0.0203	0.0163	30	74	0.0043	0.0035
18	313	0.0182	0.0146				

**Figure 2:**
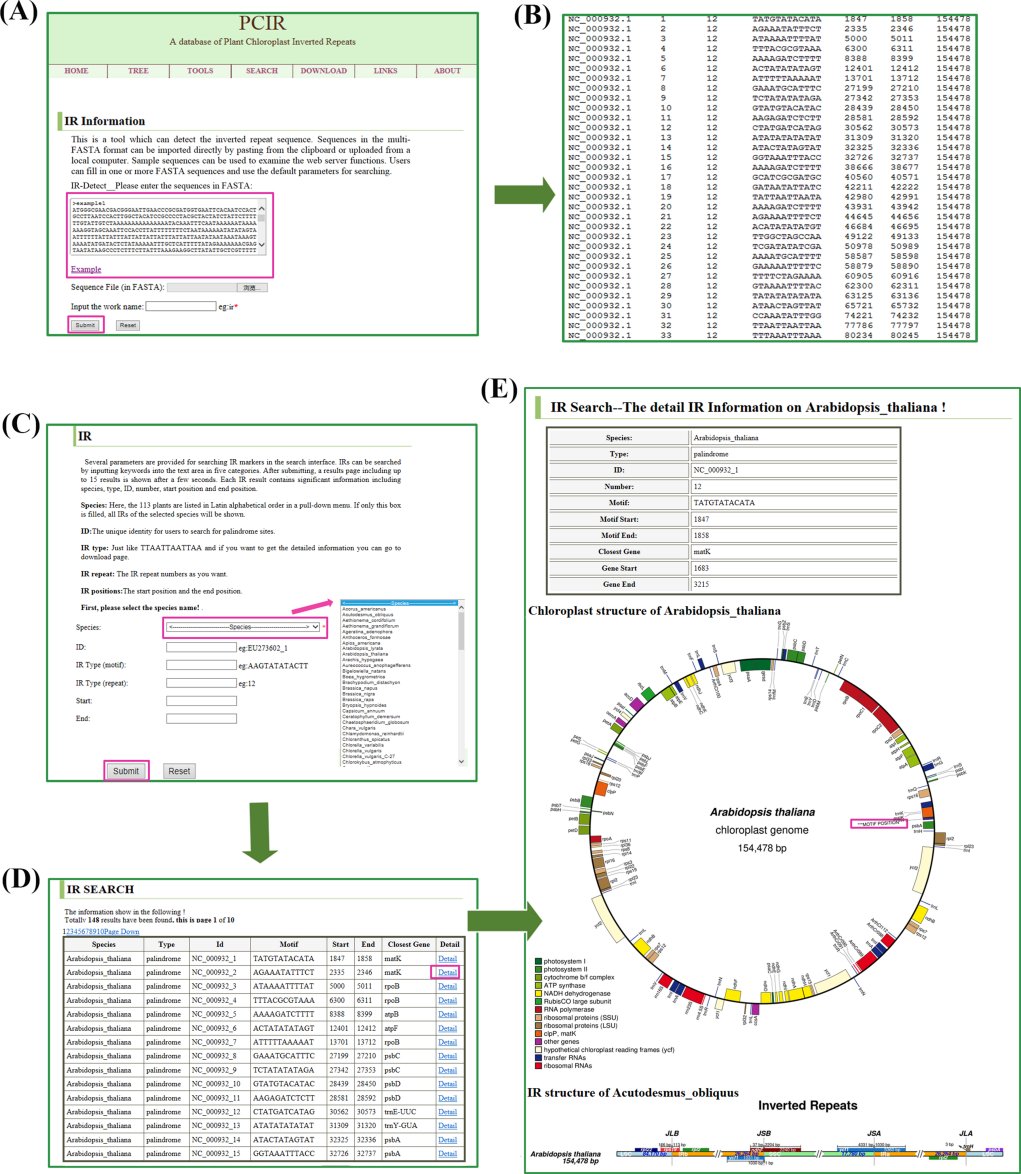
Retrieval of IR sites in the database. (**A**) The tool interfaces. The sequences in FASTA format can be filled into the test area to find IR sites. (**B**) The display of a sample tool’s results. The results contain sequence name, result number, base number, motif structure, region start, region end and sequence size. (**C**) The search interfaces. IR sites can be searched by species name, markers ID, motif structure and repeat positions. (**D**) The display of a sample search’s results. The results include species, type, ID, motif, closest genes, start position and end position. (**E**) A sample (e.g. *A. thaliana*) detail information of IRs. The results contain species, type, id, number, position, circular complete chloroplast genome map and IR regions map.

## Database content

### Distribution characteristics of IRs

A total of 21 433 IRs were characterized in the 113 species. The lengths of the chloroplast genome sequences varied from 64 335 bp in Prasinophyceae, which had only 72 chloroplast genes, to 269 044 bp in *Dunaliella salina*, which had 107 genes (Supplementary Table S1). According to motif length, the IR sequences were categorized into 25 groups. Hexamers were the most abundant type with a percentage of 33.87%. With increasing IR length, the IR distribution frequency ranged from 0.4214 to 0.0043 (/KB). This result showed that the numbers of IR sites were negatively correlated with repeat length (Table 1).

### Developing IRs online

Sequences in the multi-FASTA format can be imported directly by pasting from the clipboard or uploading from a local computer (Figure 2A). Sample sequences can be used to examine the web server functions. Users can fill in one or more FASTA sequences and use the default parameters for searching. The web application provides the results in text format. The results contain the following information: sequence name, result number, base number, motif structure, region start, region end and sequence size. All files can be downloaded (Figure 2B).

### Query interface

Several parameters are provided for searching IR markers in the search interface. IRs can be searched by inputting keywords into the text area in the following five categories (Figure 2C): (i) species names, where in the 113 plants are listed in Latin alphabetical order in a pull-down menu. If only this box is filled, all IRs of the selected species will be shown; (ii) marker ID, the unique identity for users to search for palindrome sites; (iii) IR motif (such as TATGTATACATA); (iv) IR repeat number; and (v) IR positions, including start position and end position. After submitting, a results page including up to 15 results is shown after a few seconds (Figure 2D). Each IR result contains significant information including species, type, ID, motif, closest genes, start position and end position. If more than 15 results appear in the search interface, the navigation bar at the top of the results table can be used to switch pages. The number of queried results is recorded at the top of the Search page. All query information can be downloaded in batches of ‘Markers’ from the Download webpage. More detailed information can be obtained by clicking on the details, including a visualization of the chloroplast genome and IRs.

**Figure 3:**
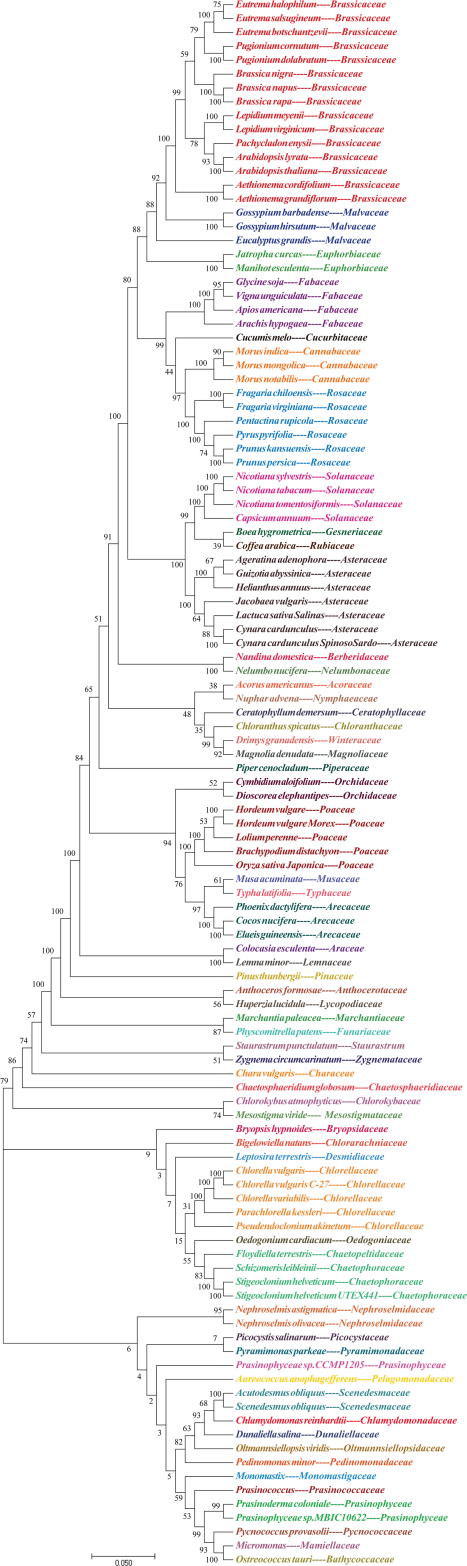
Evolutionary tree of plants. NJ phylogenetic tree reconstruction contains 113 species among 60 families based on concatenated sequences using a maximum likelihood method of all chloroplast genomes.

### Visualization of IRs

PCIR provides two ways to visualize IRs; one is by showing the circular complete genome and the other is by showing the linear regional genome. *Arabidopsis thaliana* (GeneBank: NC_000932.1) was selected as an example (Figure 2E). In the high-quality circle map, different-colored diamonds represent various structures, and the complete cpDNA genome size is marked. The inverted repeats and functional genes including rRNA, tRNA and protein-coding genes are displayed in the outer ring. In the regional map, an elaborate comparison of four junctions, JLB (LSC-IRB), JSB (SSC-IRB), JSA (SSC-IRA) and JLA (LSC-IRA), between the two single-copy regions (LSC/SSC) and two IRs (IRA/IRB) is shown and the length of each region can be observed. In *A. thaliana*, the JLB junction is covered by *rps19* and the gene *ndhF* can be seen to be 37 bp away from the IRB-SSC border. Correspondingly, JSA is located in *ycf1*, which is a pseudogene for plant viability, and the *trnH* gene is located in the LSC region, 3 bp away from the IRA-LSC border. These maps can be found in the Search page of detailed species information and can be downloaded from the Download page.

### Phylogenetic analysis and annotations

Based on the cpDNA genome multi-sequence alignments, a phylogenetic tree of individual groups is shown on the Tree webpage. The dendrogram contains 81 species in 38 families of angiosperms or gymnosperms and 32 species in 22 families of green algae (Figure 3). Selecting a species will open a new page of detailed species information from NCBI, including accession number, version, organism and features. To further explore the related genes in these chloroplast genomes, all genomes were subjected to annotation analysis. The gene information includes start position, end position, plus or minus strand and protein sequence and can be downloaded from the Download page. Related sister species had similar IR border positions, distribution architectures and gene annotations. This finding strongly supports the idea that IRs play significant roles in species evolution and can be used to examine genetic diversity.

## Conclusion and future works

PCIR is an interactive, web-based platform that enables browsing, searching and downloading of IR site information. It contains 21 433 IRs in 113 plant plastid genomes with gene annotations, protein resources and region visualization. This database of IRs will accelerate effective analyses of nucleic acid sequences and help researchers to discover plant regulatory mechanisms.

New IRs will be added from all existing species, and cpDNA sequences from new species with identified IR markers will be appended in an upgraded version. Users can offer useful notes on IR sequences such as polymorphisms, occurrence conditions and experiment results to make the information more useable.

## Supplementary Material

Supplementary_Table1_baz127

## Notes

**Database URL:** http://biodb.sdau.edu.cn/pcir/index.php
